# Prof. Sudhir Kumar Bhatnagar

**Published:** 2009

**Authors:** Surajit Bhattacharya

**Affiliations:** Editor, Indian Journal of Plastic Surgery, Senior Consultant Plastic Surgeon, Sahara Hospital, Lucknow, India. E-mail: surajitbh@yahoo.co.in


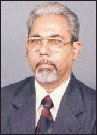


November 1, 1942 to May 27, 2009

With tears in our eyes and profound sorrow in our hearts, we the alumni of King George's Medical College and the members of Lucknow Plastic Surgery Club inform you about the sad demise of our guru, Prof. Sudhir Kumar Bhatnagar. The true fighter that he was, he fought a brave battle against stomach cancer for more than two years and breathed his last on May 27, 2009. Even during my last meeting with him, at his home, by his bedside, his enthusiasm for knowing what is new in plastic surgery had not diminished a bit.

Prof. Bhatnagar was an imaginative teacher, an innovative researcher, a meticulous surgeon and completely dedicated to his craft and his clan. To a whole generation of plastic surgeons he was a teacher par excellence, who insisted on clinical perfection as well as sound academic knowledge. Knowledge, though essential is relatively easy to acquire. He gave us the wisdom of using this knowledge, progressively, purposefully, productively, positively and profitably for ourselves and for our society. That is what our guru professed, preached and practiced. While in harness and even more, after his retirement he took a very personal interest in the development of our specialty. His work - clinical, research and academic was monumental, not only from the point of view of individual patient treatment, but also as it was instrumental in establishing the principles of treatment evolved during that time. Many of these, like inter-disciplinary collaboration while treating clefts, facio-maxillary injuries and head and neck cancers, are a norm today, but were considered bold initiatives of technology transfer, when he proposed them in those early days.

His teaching ability developed many capable plastic surgeons, who are the teachers of today. He was noted for his drive and enthusiasm. We, his students, recall how he was ready to discuss ideas with anyone – whether the highest ranking professor or a junior medical student – who showed interest. He was also noted for his ability to get down to the basic principles in surgery and his assumption that if anything can be thought out, it can be done was truly infectious. We were made to believe that once our mind stretched to a new idea, it would never goes back to its original timid and restricted dimension!

The greatest responsibility of a surgical teacher is to provide conditions for superlative work, to stimulate those working under him and to hope they will excel every time they perform. By that yardstick I feel Prof. Bhatnagar stood head and shoulder above all others. He fired in us outrageous ambitions and assured us at every step that success was round the corner. He would often say “you imagine what you desire, you will achieve what you imagine and at last you create what you dare to dream!”

In every meeting he would deservingly get all the adulation and admiration of his peers, students and junior colleagues. His brightly coloured shirts, which he wore in APSI meetings, were an embodiment of his equally multifaceted personality. A diehard member of the Lions Club, he rose to become the District Governor and his zeal for voluntary service was legendary.

Prof. S.K. Bhatnagar graduated from S.N. Medical College, Agra and did his MCh in Plastic Surgery from Safdarjung Hospital, New Delhi. He joined King George's Medical College as a Lecturer and rose to become the Professor and Head of the Department. During his teaching career, which spanned well over 30 years, he was the examiner for MCh (Plastic Surgery) in 14 universities, and was also a Member of the Curriculum Committee (Plastic Surgery) - Medical Council of India (MCI). He was instrumental in guiding 56 MCh / MS theses, including one of yours truly and was a perfectionist to the core. In the pre-computer era, with no provision for cut – paste in MS Word, I remember writing my thesis 17 times in long hand!

He had 64 research publications, 86 podium presentations in national and international conferences, and had authored six chapters in textbooks. His publications have received 371 citations in international journals of repute with one paper on Lateral Thoracic Region Flap getting 101 citations till 2007. He had helped in designing and standardizing five new flaps and that alone was a testament to his motto in life that ‘Imagination is the beginning of creation!’ He could not just imagine new and better things; he could become new and better in our eyes every day. A teacher who inspired us to become like him and then take the goalpost a bit farther every day, by setting higher and loftier standards. He, to my mind, was the most worthy recipient of the prestigious B.C Roy Award for an eminent teacher.

Prof. Bhatnagar was a past President of the Association of Plastic Surgeons of India (2000) and National Academy of Burns, India (2003). He was bestowed with *Rashtriya Chikitsak Ratan Award* (2001), Peet Prize (1987) and Gold Medal for the Best Article published in the *Indian Journal of Surgery* (1989). Knowledge may give weight but accomplishments give lustre and on this account alone, he was one of our brightest and most shining alumni. We became plastic surgeons because we idolized him and wanted to become like him – not just a good surgeon or a good teacher, but an outstanding human being.

Prof. Bhatnagar is survived by his wife Dr. Archna Bhatnagar, a senior gynaecologist in Lucknow and two sons – Ankur, a plastic surgeon and Shirish, a paediatric gastroenterologist, who will surely carry on his unfinished agenda of helping the suffering humanity. May his soul rest in peace!

